# A comparative study of Venezuelan immigrants’ pre- and post-migration concerns for their children in the United States and Colombia

**DOI:** 10.1371/journal.pone.0313215

**Published:** 2024-12-23

**Authors:** Aigerim Alpysbekova, Pablo Montero-Zamora, Mary H. Soares, Carolina Scaramutti, Sumeyra Sahbaz, Maria Duque, Tara Bautista, Maria Fernanda Garcia, Christopher P. Salas-Wright, Mildred M. Maldonado-Molina, Melissa M. Bates, Augusto Pérez-Gómez, Juliana Mejía Trujillo, Eric C. Brown, Seth J. Schwartz

**Affiliations:** 1 University of Texas at Austin, Austin, TX, United States of America; 2 Florida International University, Miami, FL, United States of America; 3 University of Miami, Coral Gables, FL, United States of America; 4 Boston College, Chestnut Hill, MA, United States of America; 5 Northern Arizona University, Flagstaff, AZ, United States of America; 6 Corporación Nuevos Rumbos, Bogotá, Colombia; 7 University of Florida, Gainesville, FL, United States of America; Public Library of Science, UNITED KINGDOM OF GREAT BRITAIN AND NORTHERN IRELAND

## Abstract

Research suggests that forced migration may lead to cultural stress and psychological distress. However, little is known about immigrant parents’ pre- and post-migration concerns for their children’s welfare. The present study examined the concerns of Venezuelan parents who migrated to the United States versus those who migrated to Colombia, and whether post-migration concerns were related to cultural stressors, mental health, and cultural identity. A sample of 609 Venezuelan immigrants completed surveys and responded to an open-ended prompt asking about pre- and post-migration concerns for children’s welfare. Lack of safety was the most common pre-migration concern for Venezuelans in the U.S., whereas lack of food was the most common pre-migration concern for Venezuelans in Colombia. More years in the destination country since arrival were linked to heightened economic concerns and reduced worries about family separation. A positive link emerged between national identity and health-related concerns for children following migration. This knowledge can inform policies and programs to better support immigrant families as they navigate the challenges of forced migration.

## Introduction

Immigration has reached its highest levels in modern history, with more than 300 million people currently residing in a country other than where they were born [1, 2]. The U.S. is home to 20 percent of the world’s migrants, and the U.S. Census Bureau reports a huge increase in the number of immigrants living in the U.S. since the 1970s [3]. The U.S. immigrant population has increased more than fourfold since 1960, currently comprising about 13.6 percent of the nation’s population (44.4 million people) [3]. The largest proportion of immigrants living in the U.S. originates from Latin American countries [4]. Although Mexicans and Cubans have traditionally represented the largest Latin American immigrant groups in the U.S., Venezuelans have recently emerged as the fastest-growing migrant group, both to the U.S. and to other Latin American countries such as Colombia [5, 6]. During the period from 1990 to 2021, the Venezuelan-origin population living in the U.S. grew from 49,000 to 660,000, increasing more than thirteenfold [6, 7]. One study [8] reports that Colombia is home to an estimated 2.8 million immigrants from Venezuela. The U.S. and Colombia are therefore two of the primary destinations for Venezuelan immigrants.

Colombia is currently home to the largest Venezuelan immigrant population anywhere in the world. This large immigrant population has created a mixed response among Colombian host nationals [9]. Some Colombians view migrants via the prism of a humanitarian response, whereas others perceive migrants as a burden on public services and a potential threat to personal and national security [10]. The large migrant influx has become a concern for the Colombian government, which has struggled to provide adequate resources to support Venezuelan immigrants. Further, research has suggested that, at least at the beginning of the Venezuelan exodus, wealthier and more highly educated individuals migrated to the U.S., whereas poorer and less educated individuals migrated to other South American countries [9]. Furthermore, historically, Venezuelan immigrants were recognized for their high levels of education and skills, with a significant proportion occupying management positions in the U.S. between 1990 and 2000 [25]. However, among crisis migrant populations that relocate to many different destination countries, educational attainment tends to be lower among those who settle in neighboring countries and higher among those settling in Western countries [11].

The Venezuelan exodus constitutes an example of crisis migration triggered by social, political, economic, and environmental factors [12, 30]. It presently represents one of the largest influxes of migrants globally, surpassing the Syrian exodus [21]. Reasons for leaving Venezuela include political corruption, government repression, high homicide rates, economic crisis, sexual and gender-based violence, and a general sense of insecurity [13, 14]. This crisis has resulted in widespread shortages of basic necessities such as food and medicine [15]. Asylum applications from Venezuela to the U.S. have increased dramatically [16] such that the number of Venezuelans applying for asylum between 2017 and 2018 was greater than the sum of the next three countries combined.

Prior to 2020, most Venezuelans who relocated to the U.S. were young and highly educated [17], whereas those who emigrated to Colombia often faced more family hardship and worked for extremely low wages [18]. Migrants to Colombia were less likely to bring their children with them, resulting in an estimated count of nearly one million children left behind in Venezuela [19, 71]. Regardless of whether they migrate together or separately, parents and their children face various challenges during migration such as marginalization and discrimination, violence exposure, limited access to healthcare services, and economic insecurity–all of which may negatively affect both parents and children [20, 21]. Migration generally affects families in different ways, often depending on whether family members migrate together, are left behind, or are separated from one another [22]. It is indeed true that Venezuelan immigrants in the U.S. tend to have higher levels of education and access to resources compared to those migrating to Colombia [23–25]. As such, they may express different priorities and concerns regarding their children’s education, healthcare, and overall well-being. For example, Venezuelan immigrants in the U.S. may prioritize access to quality education in English, whereas those in Colombia may be more concerned about their children’s access to basic necessities such as food, shelter, and healthcare [26]. Furthermore, the cultural context in the U.S. differs significantly from that of Colombia. Venezuelan immigrants in the U.S. may experience cultural stress related to adapting to a new language, customs, and social norms, which can impact their parenting practices and their children’s upbringing [27]. Additionally, the U.S. may offer different opportunities and challenges for children, such as exposure to diverse cultural experiences and educational opportunities, which may elicit distinct concerns from Venezuelan parents [28]. Therefore, there may be reason to expect that Venezuelans in the U.S. would report different concerns for their children and would experience different types and degrees of cultural stress, compared to those migrating to Colombia.

Maintaining strong family ties, respecting family values, and prioritizing childrearing are often considered as being among the most critical aspects of Latino cultural streams [29]. Not surprisingly, then, crisis-migrant parents–who are forced out of their homelands because of natural or human disasters–are likely to migrate because of current or future threats to their children [30], and much of the stress and worry these parents experience both before and following migration is likely due to concerns about their children’s welfare [31]. In turn, when parents are separated from their children during migration, parents likely experience considerable stress and worry [32]. As a result, it is critical to examine the concerns that Venezuelan migrant parents express vis-à-vis their children, both in Venezuela prior to migration and in the U.S. or Colombia following arrival.

Beyond concerns related to family separation, common post-migration concerns expressed by many immigrant parents often center around their children being unwelcomed in their new environment, such as being discriminated against or bullied in school. Immigrant youth, compared to U.S.-born youth, are more likely to experience bullying, which correlates with adverse mental health outcomes, fewer close social connections, heightened loneliness, and increased likelihood of being overweight [33]. Parents may also experience discrimination themselves: according to one study [34], immigrants from Latin America often perceive considerable discrimination in a variety of settings. Indeed, in the U.S., younger Latinos (including parents of children and adolescents) may be three times as likely to experience slurs or offensive comments about their race and ethnicity than are older Latinos [35]. Another study [36] also found elevated discrimination and negative context of reception (unwelcomeness) among Venezuelan migrant parents in Colombia. As a result, the primary goal of the present research was to test the general hypothesis that, prior to migration to the U.S. or Colombia, Venezuelan parents would mostly be worried about children’s safety, whereas after moving their main concerns would center on discrimination, racism, drug use, and bullying. Both pre- and post-migration parental concerns carry important implications for Venezuelan parents’ own mental health as well as for family dynamics [37].

We also examined the extent to which pre- and post-migration concerns would differ between Venezuelan parents migrating to the U.S. versus Colombia, and whether parents’ post-migration concerns would relate to their reports of cultural stressors, cultural identity, and mental health. Cultural stress theory [38] represents an important theoretical framework utilized to explore the influence of pre- and post-migration experiences on family functioning and mental health. Cultural stress theory enumerates several culturally related stressors, including discrimination [39] and a negative context of reception, which result in marginalization of migrants and are associated with depressive and anxiety symptoms. This theory has been validated with Venezuelan migrants to the U.S. and Colombia [40]. Results indicate that, despite the presence of considerable cultural similarities between Venezuela and Colombia, greater levels of discrimination, a worse context of reception, and more symptoms of depression were reported among Venezuelan migrants in Colombia than in the U.S. [36]. These greater levels of cultural stress were attributed to Colombia’s relative newness to international migration, as well as to previous tensions between Venezuela and Colombia [41].

More generally, cultural stress theory suggests that discrimination, negative context of reception, and other related stressors serve to communicate to immigrant groups that they are not wanted. Among Latino immigrant parents in the U.S., culturally stressful experiences have been found to predict compromised involvement and communication with their children [42]. Cultural stressors have also been found to predict impaired mental health among various groups of Latino immigrant adults [39, 43]. It is possible, then, that increased cultural stress among parents may be related to greater likelihood of certain pre- and post-migration concerns for their children.

It is also possible that immigrant parents’ connections to their heritage and destination countries (i.e., ethnic and national identity) may be linked with the concerns that they express regarding their children’s welfare. For example, parents who feel more connected to their destination countries may be most likely to characterize those countries positively–and perhaps less likely to express concerns about discrimination, racism, or other forms of exclusion. It is also possible that parents who more closely identify with their countries of origin (i.e., Venezuela) may be less likely to express pre-migration concerns about adverse conditions present in Venezuela prior to migration. Indeed, the ethnic and national identity literatures [44, 45] support these propositions.

Research specifically on parents’ concerns for their children is somewhat scarce and has focused primarily on post-migration concerns. For example, one study [46] found that many Eastern European parents in the U.S. were concerned about their children’s education. Other work [47] has focused on parents’ reasons for migrating, but these reasons often focused on the parents’ own work and educational pursuits. There is thus a need to explore immigrant parents’ concerns for their children both prior to and following migration, and to examine whether these concerns may be connected with parents’ cultural stressors, ethnic and national identity, and mental health. We pursued these objectives in the present study.

## Purpose of the present study

The present study was a mixed-method, cross-national investigation of Venezuelan migrants’ pre- and post-migration experiences. It is part of a larger project on Venezuelan migrant parents’ experiences of in the U.S. and Colombia. The purpose of our study was to examine concerns for children’s welfare among Venezuelan parents migrating to the U.S. versus those migrating to Colombia, and whether these concerns were related to cultural stress, ethnic and national identity, and mental health. As stated earlier, we hypothesized that Venezuelan immigrant parents would be concerned primarily about their children’s safety prior to migration, and about bullying, drugs, racism, and discrimination following migration. Due to a lack of prior literature, we did not advance a priori hypotheses regarding between-country differences in the specific concerns that parents would express in the U.S. versus in Colombia. We also did not advance a priori hypotheses regarding links between parents’ concerns and other study variables.

## Method

### Design and sample

Respondent driven sampling was used in this study because it is the most common probability-based method for sampling hidden or difficult-to-reach populations [48]. Within this approach, initial respondents are referred from community partners, and future respondents are reached by following the social networks of those who have already been sampled. The present study was conducted in the fall of 2017. Venezuelan immigrant adults with one or more children below age 18 were eligible to participate. Partnerships with community organizations at both sites were leveraged to facilitate recruitment. A South Florida Venezuelan community leader distributed study invitations and encouraged people to participate; and community partners in Bogotá, Colombia referred participants to our team members there. More than 90% of participants were living in the Miami or Bogotá metropolitan areas.

Consistent with migration patterns of Venezuelans to the U.S. and Colombia, length of time in the destination country differed significantly across these two countries, χ^2^(3) = 156.58, *p* < .001, Cramér’s *V* = .50. In the United States, 33.5% of participants had arrived within 1 year prior to assessment, 29.2% within 1–2 years prior to assessment, 10.6% within 2–3 years of assessment, and 26.7% more than 3 year prior to assessment. In Colombia, 80.3% of participants had arrived within 1 year prior to assessment, 15.7% within 1–2 years prior to assessment, 2.7% within 2–3 years of assessment, and 1.3% more than 3 years prior to assessment.

The vast majority (90.1%) of participants in the U.S. lived with their children, compared to 58.9% of participants in Colombia, χ^2^(1) = 64.87, *p* < .001, ϕ = .37. Participants in the U.S. were significantly older (*M* = 36.80, *SD* = 8.90) compared to those in Colombia (*M* = 31.04, *SD* = 7.96), *t(*644) = 8.63, *p* < .001, *d* = .68. Moreover, the U.S. sample was significantly more educated, χ^2^(4) = 56.17, *p* < .001, Cramér’s *V* = .30. Specifically, participants in the Colombia sample were significantly more likely not to have completed high school (16.8% versus 5.5%) or to have graduated high school but not pursued further education (18.5% versus 8.6%). Conversely, participants in the U.S. sample were more likely to have a college degree (48.9% versus 25.4%).

### Inclusivity in global research

Additional information regarding the ethical, cultural, and scientific considerations specific to inclusivity in global research is included in the S1 Checklist.

### Procedures

The University of Miami Institutional Review Board (IRB) approved the present study as protocol number 20170821.Approval was provided on October 3, 2017, and data were collected between October 9^th^ and October 20^th^. We obtained written informed consent from all adult participants involved because surveys were completed online, participants were asked to check a box if they consented to participate. Each participant was provided with a detailed online consent form outlining the study’s purpose, procedures, potential risks, and benefits. Consent forms were securely stored in accordance with ethical guidelines and privacy laws.

Community partners in Colombia were asked to refer participants to an office space in Bogotá, where participants were asked to complete an online survey. Word-of-mouth recruitment was used to enroll new participants in Colombia. After completing the survey, participants were given gift card payments for 75,000 Colombian pesos (approximately US$25). In the U.S., the first 22 participants were recruited by community leaders. The remaining 317 participants were recruited through referrals. Respondents received a $40 gift card for completing the survey and were given the option of nominating up to three eligible study participants, with an additional $15 gift card offered for each referred individual who completed the survey (for a total potential incentive of $85). Participants were instructed to complete the questionnaire online using a link provided by the research team. Payment differences between the U.S. and Colombia reflect the differing value of the U.S. dollar across the two countries. The IRB’s from the University of Miami and Boston College, as well as the ethics committee from Corporación Nuevos Rumbos in Bogotá, approved this study. Participants provided informed consent prior to participating in the study. Participants were informed of their right to withdraw from the study at any time and were assured that their responses would be kept confidential. No identifying information was collected.

### Measures

Participants were asked to complete the survey questions electronically using Qualtrics. Following a back-translation procedure [49], survey questions were administered in Spanish. Two bilingual researchers from Bogotá and Miami collaborated on back translating the English version of the survey into Spanish. These same translators then back translated the open-ended responses from Spanish into English.

#### Discrimination

We used the 7-item Perceived Discrimination Scale (PDS-7) to assess perceived discrimination [50]. This scale assesses the degree to which individuals feel they have been treated unfairly or negatively based on their nationality or ethnicity. Items inquire about being viewed with suspicion or as inferior due to one’s background. A sample question is: “*Do people you do not know treat you unfairly or negatively because you are Venezuelan*?” Cronbach’s alpha was .91 in the U.S. and .86 in Colombia.

#### Negative context of reception

The 6-item Negative Context of Reception Scale [40, 51] was utilized to measure the extent to which individuals feel that they are not welcome in their new country due to their nationality or ethnicity. A sample item is "*People from Venezuela are not welcome here*." Cronbach’s alpha was .87 in the U.S. sample and .85 in the Colombia sample.

#### Anxiety

We used the General Anxiety Disorder (GAD-7) scale [52] to assess anxiety symptoms. This scale consists of 7 items designed to measure how often individuals experience anxiety symptoms such as feeling nervous, anxious or on edge, and not being able to stop or control worrying. A sample item is “*Worrying too much about different things*” during the week prior to assessment. Cronbach’s alpha was .89 in the U.S. and .87 in Colombia.

#### Depressive symptoms

To assess depressive symptoms, we used the 10-item Boston form of the Center for Epidemiological Studies Depression Scale (CESD-B) [53, 54]. This scale assesses how often individuals experience symptoms of depression such as sadness and lack of energy. A sample item is "I felt like everything I did require a lot of effort.". Cronbach’s alphas were .89 in the U.S. and .88 in Colombia.

#### Ethnic identity

The seven affirmation/belonging items from the Multigroup Ethnic Identity Measure (MEIM) [50] were used to assess ethnic identity. Sample items include “I am proud to be Venezuelan.” Cronbach’s alphas were .89 in the U.S. and .87 in Colombia.

#### National identity

The seven affirmation/belonging items from the American Identity Measure (AIM) [55] were adapted to assess the exploration of national identity for people in U.S. or Colombia. The AIM consists of 12 items and has been validated across ethnic group and immigrant generation. Cronbach’s alphas in the present sample were .94 in the U.S. and .97 in Colombia. Sample items include “I am proud to be American/Colombian.”

#### Concerns for children

We included two open-ended prompts to elicit parents’ concerns for their children’s welfare both before and following migration. The prompts read as follows: “As parents, we often have concerns about our children’s well-being and development. Concerns may relate to the safety of your neighborhood, the quality of your children’s schools, your children’s friends and cultural influences, or many other things. In the spaces below, please describe the concerns, if any, you have for your children. (1) Before you left Venezuela, what did you worry about for your children? (2) Since moving to the US/Colombia, what do you worry about for your children?”

### Data analysis

We used a sequential mixed method approach where the qualitative component informed the outcomes (i.e., categories and sub-categories) to be analyzed in as part of a subsequent quantitative phase. In phase 1 (i.e., the qualitative portion of the study), a codebook was created to identify the categories and sub-categories emerging from the data. The codebook was created using guidelines for content analysis [54]. Categories and sub-categories were identified through a process of iterative coding, where data were reviewed multiple times to identify patterns and connections between codes. Three authors (A.A., M.S., and S.J.S.) performed all coding tasks independently and met on several occasions to discuss disagreements. Between meetings, each author coded the data multiple times to identify patterns and connections between codes, and coding decisions were then discussed among the three coders until they reached complete agreement. Using this approach, we developed a final codebook for parents’ concerns about their children (a) before and (b) after leaving Venezuela. The codebook includes two main questions, eight categories, and 19 sub-categories. To ensure reliability and validity of the coding process, we calculated the percentage agreement among the three coders. This was done by comparing the codes assigned by each author to the same segments of data. The percentage agreement was calculated as Number of Agreements/(Number of Agreements + Number of Disagreements)*100. The initial percentage agreement on these codes (prior to discussing disagreements) was adequate (85.7%). Final codes were then transferred to an Excel sheet to validate the highest-level sub-categories against the parents’ responses.

As part of the quantitative phase (i.e., Phase 2), all codes were converted into dichotomous variables (Yes = 1, No = 0) to indicate whether each participant endorsed a specific concern. Then, we computed descriptive statistics to examine the prevalence and distribution of each pre- and post-migration concern using M*plus* version 8.8. Next, we explored the associations of each type of concern with ethnic identity, national identity, cultural stressors, and mental health among Venezuelan immigrants. Specifically, we conducted 14 different probit regressions (six for pre-migration concerns and eight for post-migration concerns) to determine the associations of concern-related categories with quantitative variables. Within each probit model, we controlled for age, sex, marital status (married versus other), educational attainment (college graduate or more versus not), country of arrival (Colombia versus US), and years since arrival to the destination country (e.g., if year of arrival was 2000, then the number of years was 17). These control variables were included to account for demographic and socio-economic differences that could influence participants’ concerns. Indeed, except for marital status, all covariates emerged as significant predictors of at least one of the outcomes. We retained all of the covariates, however, because married or partnered parents likely differ from single parents on a number of parenting variables [56].

## Results

### Phase 1: Qualitative findings

Because the majority of participants wrote their concerns as single words or phrases (e.g., insecurity, going hungry, drug use at school), we summarize these responses here in text rather than providing specific participant quotes. As a first step of qualitative analysis, we examined the frequency with which each category and sub-category was represented in the data. Sub-categories that were mentioned extremely infrequently (only two or three times), such as "Other," "Fear of the Future," "No Concerns," and "Lack of Access to English Classes," were eliminated from further analysis. The remaining pre-migration sub-categories were (a) health-related issues, (b) lack of food, (c) lack of education, (d) lack of safety, (e) lack of finances, and (f) lack of freedom (See **S1 Appendix**). Remaining post-migration sub-categories included (a) drug use at school, (b) legal services, (c) health-related issues, (d) lack of education, (e) lack of finances, (f) family separation, (g) bullying at school, and (h) lack of friends (See **S2 Appendix**).

The supporting information provided in **S1 Table** presents the concerns reported by participants in the U.S. and Colombia at both pre- and post-migration. The sample size was 609, with 311 participants in the U.S. and 298 in Colombia (38 participants did not answer the question about concerns, or provided responses that could not be aggregated into sub-categories). Overall, before migration, the most common concerns were: (1) lack of safety (49%), such as “Security for my son, the place where we lived was unsafe”; (2) lack of education (39%), such as “The stability and ease of my daughter, the education was low, the teachers abandoned their work to find food and the children would go and not have class, and insecurity”; and (3) lack of food (32%), such as “I couldn’t find food for my family especially my son.”

Within countries, the most prevalent pre-migration concern reported by Venezuelans in the U.S. was lack of safety (59%), such as “Insecurity and especially for the children, they robbed us, they pointed at my children with guns”, whereas in Colombia the most prevalent pre-migration concern was lack of food (53%), such as “Shortage of food made us make the decision.” Except for concerns related to lack of education, prevalence rates for all pre-migration concerns differed significantly between the two study sites.

The most common post-migration concerns in the full sample were (1) lack of education (19%), such as “Delinquency of a communist government, corruption, lack of food and medicine, communist socialist Cuban education”, (2) family separation (16%), such as “due to the great immigration because of the current government there are many separated families and people that are still in Venezuela who are repressed by the current government” and “I was separated recently and came to Colombia and I am worried that my children will forget about me or stop loving me,” and (3) drug use at school (15%), such as “new friendships, there are many kids here with earrings and taking drugs, and their influence worries me” and “relationships with people, influence, there is more addiction to drugs, violence and insecurity.”

Within countries, the most prevalent post-migration concern reported by Venezuelans in the U.S. was drug use at school (14% of the U.S. sample), whereas in Colombia the most prevalent concern was family separation (30% of the Colombia sample). Again, the differences between the two groups were statistically significant for most concerns, except for drug use at school and lack of friends (see **S1 Table**).

### Phase 2: Quantitative findings

**S2 Table** presents the results of a series of probit regressions examining the factors associated with probabilities of *pre-migration* concerns. In terms of pre-migration concerns, a statistically significant positive relationship between age and lack of safety also emerged (β = .154, *p* < .01). Furthermore, having a college degree or higher was significantly and positively associated with reporting a lack of safety (β = .164, *p* < .01). Regarding differences between destination countries, compared to those who migrated to the U.S., immigrants who migrated to Colombia reported significantly greater concerns related to health issues (β = .413, *p* < .001), lack of food (β = .444, *p* < .001), economic issues (β = .298, *p* < .01), and fewer concerns about lack of freedom (β = -.204, *p* < .05). Venezuelan participants who reported a longer duration of residence in their destination country (US versus Colombia) indicated fewer pre-migration concerns about lack of food (β = -.252, *p* < .01).

Ethnic identity was not associated with any of the pre-migration concerns. However, national identity was significantly and positively associated with reporting concerns about health issues (β = .153, *p* < .01). Interestingly, discrimination was not a significant predictor of any of the pre-migration concerns. However, a statistically significant relationship emerged between negative context of reception and reporting concerns about lack of education (β = .158, *p* < .05). Additionally, depressive symptoms were significantly associated with lower educational attainment (β = -.187, *p* < .01) and with concerns about lack of safety (β = .222, *p* < .01) and about economic issues (β = .264, *p* < .01). No significant associations emerged between anxiety and pre-migration concerns.

**S3 Table** presents associations between quantitative variables and the likelihood of reporting a specific *post-migration* concern. Pre-migration health-related issues were positively associated with post-migration health related issues (β = .189, *p* < .01). Pre-migration concerns about lack of food were positively associated with post-migration concerns about family separation (β = .099, *p* < .05). Concerns about economic issues before migration were positively associated with family separation concerns after migration (β = .083, *p* < .01). Concerns about lack of freedom before migration were negatively associated with concerns about health-related issues after migration (β = -.151, *p* < .05). Age was positively associated with reporting concerns about drug use at school following migration (β = .204, *p* < .01), but age was negatively correlated with post-migration concerns about health issues (β = -.266, *p* < .05).

Results also indicate that fathers were less likely than mothers to report post-migration concerns about drug use at school (β = -.169, *p* < .05) and about health issues (β = -.159, *p* < .05). On the other hand, fathers were more likely than mothers to report post-migration concerns related to family separation (β = .107, *p* < .05).

We also found a significant correlation between migrating to Colombia and reporting post-migration concerns about legal services access, lack of education, economic issues, and family separation, with beta coefficients ranging from .197 to .422 (all *p*s < .05). Regarding years since arrival to the destination country (US versus Colombia), participants who reported more years in the new country also reported more concerns about economic issues (β = .161, *p* < .05). In contrast, more years since arrival were associated with fewer concerns about family separation (β = -.430, *p* < .001). In addition, we found a significant negative association between depressive symptoms and the probability of reporting concerns about drug use at school (β = -.224, *p* < .05). Finally, we found a positive correlation between depressive symptoms and the probability of reporting concerns related to family separation (β = .135, *p* < .05).

To further explore the relationship between pre-migration concerns and post-migration outcomes, we estimated an additional model where pre-migration concerns were included as predictors of post-migration concerns. Model fit indices indicated a good fit to the data: χ^2^ (72) = 84.835, *p* = .14, CFI = .981; RMSEA = .025 (90% CI = .000 to .043). This model also allowed us to examine the extent to which how pre-migration concerns would influence post-migration concerns differentially within the US and Colombia groups. The numbers presented in **S3 Table** are for the sample as a whole–here we present the results separately for the US and Colombia samples.

We found two “autoregressive” effects in the US group–that is, effects where a pre-migration concern predicts the same concern after migration–and two additional predictive effects involving pre-migration and post-migration concerns. In the US group, autoregressive effects were observed where pre-migration health-related concerns significantly predicted health-related concerns post-migration, β = .215, *p* = .024. Additionally, concerns about lack of education prior to migration significantly predicted similar concerns after migration, β = .295, *p* = .001. In the Colombia group, significant associations were found between pre-migration concerns about lack of food and post-migration concerns about lack of education (β = -.170, *p* = .030) and concerns about being separated from family members (β = .193, *p* = .011).

## Discussion

Our findings concerning Venezuelans’ pre- and post-migration concerns in Colombia and the U.S. suggest that complex socioeconomic, cultural, and political factors play a significant role in shaping those concerns. Our results indicate that the concerns of Venezuelan parents before and after migration to Colombia and the U.S. are influenced by a range of complex socioeconomic, cultural, and political factors. Below we highlight several key findings.

The first key finding is that the pre- and post-migration concerns expressed by Venezuelan immigrants differ significantly across destination countries and may be related to cultural stress, mental health, and national identity. For instance, the most common pre-migration concerns within the entire sample were lack of safety, lack of education, and lack of food in Venezuela. Our findings align with one study [57] highlighting the prevalence of pre-migration hunger among Venezuelan immigrant adolescents in the United States. Additionally, within-country comparisons indicated that Venezuelans residing in the U.S. were most likely to express concerns about their safety prior to migration, whereas those Venezuelans who lived in Colombia were most likely to express concerns related to health issues, lack of food, and economic issues (and were less likely than those in the U.S. to mention lack of freedom). These findings suggest that Venezuelan immigrants in Colombia experienced harsher socioeconomic conditions in Venezuela compared to those who migrated to the United States. These results are in line with one study [58], who reported that Venezuelans in Colombia faced many obstacles, such as lack of food and healthcare, prior to migration. Compounding these more severe and numerous pre-migration concerns is the inability of Colombia, as a receiving country, to meet the needs of newly arrived migrants. Especially at the time these data were collected, migration to Colombia was a far newer phenomenon among Venezuelans than is migration to the United States, as evidenced by the far more recent arrivals of our Colombia participants compared to our U.S. participants. Many Venezuelans in Colombia were homeless and lived in precarious tent encampments, where they are vulnerable to hygiene-related diseases and crime [59]. Despite the Colombian government’s response to the immigrant influx, including the establishment of homeless shelters, challenging economic conditions in Colombia increased the difficulties for Venezuelans with lower levels of education vis-à-vis securing jobs that offered sufficient income to meet basic needs.

At least at the time these data were collected, Venezuelans residing in the U.S. were more likely to live in houses or apartments, either independently or with relatives, primarily due to their higher education levels and employment opportunities. However, Venezuelans migrating to the United States prior to 2020 were often those who worked in professional occupations and had been targeted for criticizing or opposing the Maduro regime [17]. Our finding that longer residence in the destination country is associated with fewer reported pre-migration concerns about lack of food can be understood through the lens of the "immigrant optimism hypothesis” [60]. This hypothesis suggests that, as migrants spend more time in the destination country, they adapt more, and their perceptions of past hardships diminish. Similarly, one study [61] found that a balanced time perspective correlates with better psychological adaptation, suggesting that over time, migrants may develop a more integrated view of their past, present, and future, which could include a deeper understanding of past hardships. Another study [62] discusses the stereotype accommodation hypothesis, which implies that immigrants’ preexisting beliefs, including those about past hardships, may change as they adapt to the destination culture, but this process is influenced by the length of stay and acculturation orientation.

Our finding that pre-migration health-related issues are positively associated with post-migration health-related issues aligns with previous research indicating that health problems experienced prior to migration can worsen after migration due to the added stress of adapting to a new environment and potential lack of access to adequate healthcare in the destination country [63]. Indeed, health-related concerns are often compounded by the challenges of navigating a new healthcare system, language barriers, and potential discrimination, all of which can hinder effective treatment and support [64]. The positive association between pre-migration concerns about lack of food and post-migration family separation concerns suggests that pre-migration food insecurity may heighten anxieties about maintaining family cohesion post-migration. This may be due to the psychological strain and resource scarcity experienced before migration, which can carry over and influence perceptions of family stability and support in the destination country [65]. One study [66] examined the impact of conflict and relocation on Ukrainian refugee mothers in Europe, noting that cultural perceptions of child nutrition and challenges in accessing nutritious food during conflict and post-relocation can influence family stability and support systems in the destination country.

Our finding that economic issues before migration were positively associated with family separation concerns following migration highlights the financial strain that often accompanies the migration process, especially for forced or crisis migrants. Economic instability often necessitates migration as individuals and families seek improved financial conditions, which can result in prolonged periods of family separation. This phenomenon is evident in the context of transnational families from Latin America, such as examples where migration to Spain and subsequently to the UK was used as a strategy to achieve economic stability despite the cost of family separation [67]. Similarly, a study of refugees resettled in Australia [68] found that many experienced family separation, which significantly compromised health outcomes–suggesting profound effects of economic-driven migration on family separation. Interestingly, our finding that concerns about lack of freedom before migration were associated with fewer post-migration health-related issues concerns may suggest that individuals who perceive themselves as trapped prior to migration may experience a psychological benefit after migration, perhaps mitigating health concerns that are often exacerbated by restrictive environments.

The finding that more years in the destination country are associated with increased concerns about economic issues is supported by prior findings [69] that immigrants often encounter significant barriers to employment in their desired occupations, leading to a misallocation of the workforce and potential productivity losses in the host country. Indeed, downward economic mobility may require highly educated or skilled migrants to work in less advanced occupations following migration [70].

The most common post-migration concerns for Venezuelan immigrants were lack of education, family separation, and drug use in school. Venezuelan parents in the U.S. expressed greater concern about drug use in school, whereas Venezuelan parents in Colombia expressed greater concern about family separation. Indeed, at least prior to 2020, many Venezuelans in Colombia migrated without (or separately from) their children [71]. On the other hand, higher rates of substance use in the U.S. may lead parents living in the U.S. to worry more that their children will associate with substance-using peers. This finding aligns with existing research [40, 57] suggesting elevated rates of alcohol use among Venezuelan youth in the U.S., with cultural stress serving as a contributing factor.

Overall, our findings suggest that, whereas pre-migration concerns were primarily focused on basic needs (health issues, lack of food, economic issues, and lack of freedom), post-migration concerns are more complex and diverse (lack of education, family separation, and drug use in school). Indeed, lack of basic staples (e.g., food, clean water) and threats to personal safety are among the principal reasons why Venezuelans have left their homeland [9]. Venezuelans’ varying pre- and post-migration concerns may be explained by the vastly different socioeconomic environments to which they migrate (the U.S. versus Colombia), as well as their own experiences in Venezuela prior to migration (such as political turmoil, socioeconomic insecurity, and humanitarian crises) [2]. Consistent with the present findings, one study [39] found, that Venezuelan migrants in the U.S. moved primarily due to violence and government repression, whereas Venezuelan migrants in Colombia moved primarily due to lack of food and other basic supplies.

The positive correlation between migration to Colombia and concerns around access to legal services suggests that migrants in Colombia faced challenges vis-à-vis legal representation, advice, or protection. Despite being eligible for temporary legal status that allows them to work in Colombia, Venezuelan migrants often struggle to obtain legal advice and representation needed to access these resources [72]. The direct relationship between migration to Colombia and concerns with lack of education suggests that migrants may face challenges in accessing educational services or resources for their children. This finding is consistent with one study [73] identifying inclusion of a large number of Venezuelan immigrant students into the Colombian educational system as one of the most prominent challenges to Colombian efforts to welcome Venezuelan newcomers. Integrating Venezuelan immigrants and their children into Colombian society entails adapting current strategies for use with a new population with diverse social, ethnic, religious, economic, and cultural backgrounds–a challenge that may be daunting for low/middle income countries, such as Colombia, to meet.

The positive correlation between migration to Colombia and concerns with economic issues suggests that migrants may face greater challenges in accessing economic opportunities or resources than will people who migrated to the United States. This finding may be explained in light of the disparities between migrating to a high-income country versus a low/middle income country. Further, economic concerns among Venezuelans in Colombia may be exacerbated by the depreciation of the bolivar (the Venezuela’s official currency), which raises the cost of living in Colombia relative to its neighbor [74]. It is noteworthy that the Venezuelan currency’s value has diminished to the extent that the Venezuelan inflation rate was over 1 million percent in 2018 before the bolivar was devalued. The negative association between depressive symptoms and concerns with drug use at school suggests that parents who report higher levels of depression may be less attentive to what is happening at their children’s school. Indeed, one study [75] found that parental monitoring and supervision of adolescent activities may not be effective for parents who are high in depressed mood.

The positive correlation between depressive symptoms and concerns with family separation suggests that being separated from one’s children may increase parental depressive symptomatology. This finding is consistent with previous research [76] suggesting that post-migration family separation was linked with emotional distress and depressive symptoms among Venezuelan immigrants in Peru. Future research should examine the long-term effects of migration on mental health outcomes, as well as the effectiveness of interventions aimed at addressing immigrants’ pre- and post-migration concerns.

The associations of the identity and cultural stress variables with parental concerns may be somewhat difficult to interpret. The association between identifying with the U.S. or Colombia and reporting concerns about children’s health in Venezuela prior to migration may reflect a motivation for parents to migrate away from Venezuela to protect their children’s health. The link between negative context of reception in the U.S. or Colombia and pre-migration concerns about lack of education in Venezuela may reflect a belief that less educated families may be less likely to be welcomed in the family’s destination country. Interestingly, identity and cultural stress variables were not significantly associated with any of the post-migration concerns that parents expressed about their children’s welfare.

### Intervention and policy recommendations

Our findings suggest an interplay among socioeconomic, cultural, and political factors in shaping the pre- and post-migration concerns of Venezuelan immigrants in Colombia and the United States. The variation in these concerns between destination countries, as well as the shift from primarily basic needs prior to migration to multifaceted post-migration concerns appear to call for tailored interventions and support structures.

One critical avenue for intervention involves the implementation of cultural sensitivity training programs. Such training can provide psychoeducation to help Venezuelan migrant parents better understand and cope with the cultural nuances and differences and potential biases they may encounter in their new destination countries. Additionally, teaching individuals effective coping strategies is essential: coping strategies help people to manage the stress, anxiety, and depressive reactions that can occur in response to experiences of discrimination. Additionally, encouraging cultural identity exploration may help to foster a strengthened sense of self and, in turn, greater resilience against discrimination.

The positive correlation between migration to Colombia and concerns about legal services access, lack of education, and economic issues suggests challenges in accessing essential services. To address these concerns, policymakers and service providers in Colombia might consider strategies to increase access to legal resources and thereby facilitate integration into the educational system, and address economic disparities. On the other hand, in the United States, where parents were more concerned about exposure to drug-using peers in school, interventions may need to be provided to increase parental involvement in schooling, increase adolescents’ substance use refusal skills, and provide parents with critical knowledge and skills regarding the role of peers in children’s and adolescents’ lives in the Unites States.

## Limitations and future directions

The present findings should be interpreted in light of several limitations. First, although the current study was the first to examine Venezuelan parents’ concerns for children across multiple countries, we used cross-sectional convenience sampling to recruit and assess participants. This design limits our ability to establish directional or causal relationships between variables, and as a result we can only draw conclusions about associations. Second, convenience sampling introduces selection bias, compromising the representativeness of the sample and its ability to accurately reflect the larger population. Third, our study did not investigate the long-term effects of migration on mental health–and it is therefore essential to conduct prospective longitudinal studies with Venezuelan immigrant parents and their children. Fourth, we only sampled from Miami and Bogotá, which were home to the largest Venezuelan immigrant communities in the U.S. and Colombia, respectively, at the time of data collection. It is critical to investigate other areas in the U.S. and Colombia where Venezuelan immigrants live because perceptions of discrimination and of children’s social contexts may differ. Fifth, we only interviewed parents and did not assess the children themselves. Children may have expressed different concerns than their parents expressed. Sixth, pre-migration concerns were assessed retrospectively, whereas post-migration concerns were assessed currently. Parents may have forgotten about or otherwise misremembered some of their pre-migration concerns after arriving in their destination country. Seventh, we did not ask about legal status in the present study–which is problematic given that temporary protected status had not been granted to Venezuelans in either the U.S. or Colombia at the time of data collection. Eights, although non-immigrant Venezuelans living in Venezuela could serve as a baseline for comparisons, our primary aim was to explore the unique experiences and concerns of Venezuelan immigrants who face distinct challenges related to migration. Future research should consider including non-immigrant Venezuelans as a comparison group to provide a more comprehensive understanding of the differences between immigrants and non-immigrants. Finally, differences in data collection techniques employed in the U.S. and Colombia could have influenced the demographic differences observed between the two samples. A survey link sent via email by a community leader, for example, may attract more technologically advanced participants than an invitation to visit a storefront office.

## Conclusion

Despite these and other limitations, our study highlights the concerns of Venezuelan immigrant parents in the U.S. and Colombia before and after migration. Findings indicate that complex socioeconomic, cultural, and political factors play a significant role in shaping those concerns. Whereas pre-migration concerns focused on basic needs such as food, shelter, and safety, post-migration concerns were more complex and diverse. Age, education, and country of origin emerged as significant predictors of post-migration concerns. Our findings underscore the need for policymakers and practitioners to develop and implement effective interventions to address the unique pre- and post-migration concerns of Venezuelan immigrants in multiple destination countries. Such efforts are important both in addressing children’s needs and in potentially helping to offset cultural stress, internalizing symptoms, and identity-related challenges among parents. We hope the present study will inspire further work in this direction.

## Supporting information

S1 Checklist Inclusivity in global research. (DOCX)

S1 Table Descriptive statistics of reported concerns in the U.S. and Colombia. (TIF)

S2 Table Pre-migration reported concerns associated factors among Venezuelan immigrants. (TIF)

S3 Table Post-migration reported concerns associated factors among Venezuelan immigrants. (TIF)

S1 Appendix Codebook of the Pre-migration concerns of Venezuelan immigrants in the U.S. and Colombia. (TIF)

S2 Appendix Codebook of the post-migration concerns of Venezuelan immigrants in the U.S. and Colombia. (TIF)

S1 Data (ZIP)
