# Supplementary material for: A comparative study of Venezuelan immigrants’ pre- and post-migration concerns for their children in the United States and Colombia
Source: PLoS One. 2024 Dec 23;19(12):e0313215. doi: 10.1371/journal.pone.0313215 (PMC11665991; doi:10.1371/journal.pone.0313215)
Supplement: S1 Checklist — (DOCX) [file pone.0313215.s001.docx]

Inclusivity in global research

PLOS’ policy on inclusivity in global research aims to improve transparency in the reporting of research performed outside of researchers’ own country or community and ensures that PLOS publications reporting global research adhere to high standards for research ethics and authorship. Authors of relevant research articles may be asked to complete the questionnaire below, which outlines ethical, cultural, and scientific considerations specific to inclusivity in global research. This questionnaire may be requested when researchers have travelled to a different country to conduct research, if research uses samples collected in another country, research with Indigenous populations or their lands, or if research is on cultural artefacts. Researchers travelling to another country solely to use laboratory equipment will not normally be required to complete the questionnaire. However, the questionnaire can be requested at the journal’s discretion for any submission – if you have been requested to complete this questionnaire by the PLOS journal you submitted to, please do so.

Please complete the questionnaire below and include this as a Supporting Information file with your manuscript. Note that if your paper is accepted for publication, this checklist will be published with your article in the supporting information files. Please ensure that you reference the checklist in the main body of your manuscript. We suggest adding a subsection ‘Inclusivity in global research’ to your Methods section and adding the following sentence: “Additional information regarding the ethical, cultural, and scientific considerations specific to inclusivity in global research is included in the Supporting Information (SX Checklist)”

The questions have been designed to be applicable to a wide range of study types, and there are subsections for both human subjects research and non-human subjects research. If any of the questions are not relevant to your research please mark them as “N/A” as appropriate.

**S1 Checklist.** **Ethical considerations, permits and authorship**

*This section is applicable to all research types.*

Provide details as to who granted permissions and/or consent for the study to take place in the Methods section of your manuscript. This should include the names of **all** ethics boards, governmental organizations, community leaders or other bodies that provided approval for the study. If individuals provided approval refer to these people by their role or title but do not list their name(s).

Reported on page number: pages 9-11

If there were any deviations from the study protocol after approval was obtained please provide details of these changes in the Methods section of your manuscript.

Reported on page number: N/A

Did this study involve local collaborators that are residents of the country where the research was conducted or members of the community studied? If you do not have any authors from said communities, please provide an explanation for this below.

Founder of the Raices Venezolanas Miami (Venezuelan Roots) Patricia Andrade helped us in recruiting participants for the U.S. sample.

Everyone listed as an author should meet PLOS’ criteria for authorship and all individuals who meet these criteria should be included in the author byline, rather than the acknowledgements. For further information please see the journal’s Authorship Policy.

**Human subjects research (e.g. health research, medical research, cross-cultural psychology)**

Did you obtain written informed consent from a representative of the local community or region before the research took place? How did you establish who speaks for the community? Details of written informed consent obtained from study participants should be reported separately in the Methods section of your manuscript.

Yes, we obtained written informed consent from a representative of the local community in Columbia and U.S. before the research took place. To establish who speaks for the community, we engaged in preliminary meetings with local leaders to identify an appropriate representative.

How did members of the local community provide input on the aims of the research investigation, its methodology, and its anticipated outcome(s)?

When engaging with the local community, how did you ensure that the informed consent documents and other materials could be understood by local stakeholders?

We used a back-translation procedure to translate the survey questions and informed consent documents into Spanish. Two bilingual researchers from Bogotá and Miami collaborated on this process, ensuring that the translations were accurate and culturally relevant. The research teams were proficient in both Spanish and English.

Community partners in Colombia were asked to refer participants to an office space in Bogotá, where participants were asked to complete an online survey*.* Word-of-mouth recruitment was used to enroll new participants in Colombia. A South Florida Venezuelan community leader distributed study invitations and encouraged people to participate

Will the findings of the research be made available in an understandable format to stakeholders in the community where the study was conducted (e.g. via a presentation, summary report, copies of publications, etc.)? Please provide details of how this will be achieved.

Copies of the publications resulting from the research will be made available to the community. We will provide printed and digital versions of the papers, ensuring that they are accessible to all interested stakeholders.

**Non-human subjects research using specimens/ animals collected as part of the study, or those housed in archival collections. Examples include archaeology, paleontology, botany and zoology.**

Did the permission you obtained from a local authority to perform the study include an agreement on access to outputs and benefit sharing? This may include procedures to enable fair distribution of the benefits and resources arising from the research performed. Please include any details of Prior Informed Consent and Benefit Sharing Agreements obtained. These may be required by field-specific regulations, for example the Convention on Biological Diversity (CBD) and the associated Nagoya Protocol.

The study was conducted in compliance with ethical standards and regulations. The Institutional Review Boards (IRBs) of the University of Miami and Boston College, as well as the ethics committee from Corporación Nuevos Rumbos in Bogotá, reviewed and approved the study protocols. Before commencing the study, we obtained Prior Informed Consent (PIC) from local authorities and community leaders. This process involved thorough discussions with the community to explain the study's purpose, methodology, potential risks, and benefits. The community leaders provided their consent based on a clear understanding of the study and its implications. Participants received financial incentives for their participation, as well as additional compensation for referring other eligible participants.

If the material used in your study was imported, please A) provide the year it was imported and B) indicate whether permits were obtained to import/export the materials used, C) provide details of any permits obtained. If this information is not available, please indicate this.

The study did not involve the importation or exportation of physical materials. The research primarily utilized online surveys and digital data collection methods, which did not require the transport of physical goods across borders

If you used archival specimens, please state how the material used in your study was acquired by the institute it is held in and provide details of any permits obtained for the original excavations/ sample collection. If this information is not available, please indicate this.

The study did not involve the use of archival specimens.

How was the potential cultural significance of the materials collected in your study to local communities considered in your research design? Were Indigenous peoples and/or local researchers and institutions involved with archaeological excavations / collection of specimens? If so, please provide a description of their involvement.

N/A

If your manuscript includes photographs of human remains please indicate whether authors obtained permission from descendants or affiliated cultural communities to do so.

N/A
