# Supplementary figures and images for: A comparative study of Venezuelan immigrants’ pre- and post-migration concerns for their children in the United States and Colombia
Source: PLoS One. 2024 Dec 23;19(12):e0313215. doi: 10.1371/journal.pone.0313215 (PMC11665991; doi:10.1371/journal.pone.0313215)

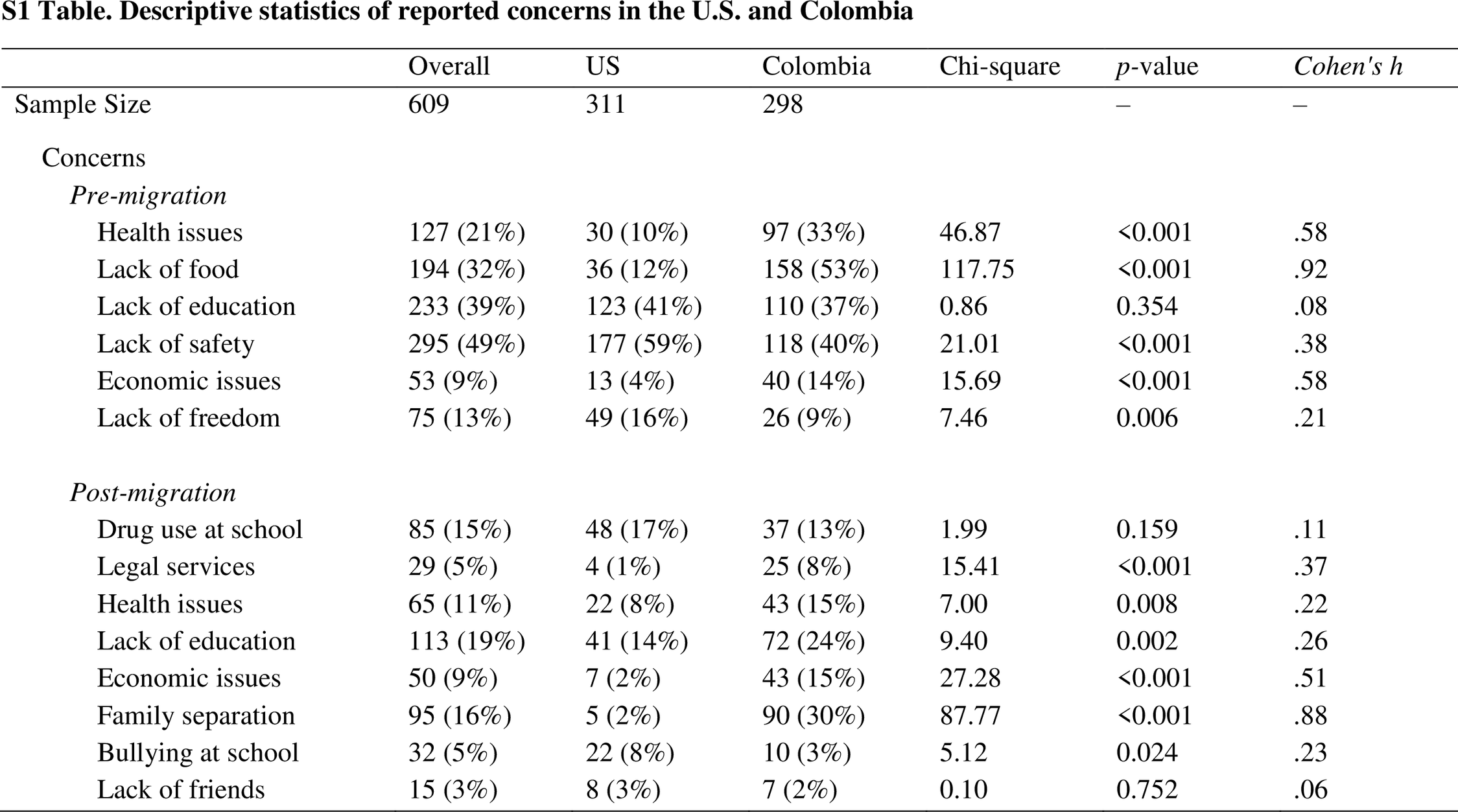

Supplement: S1 Table — (TIF) [file pone.0313215.s002.tif]

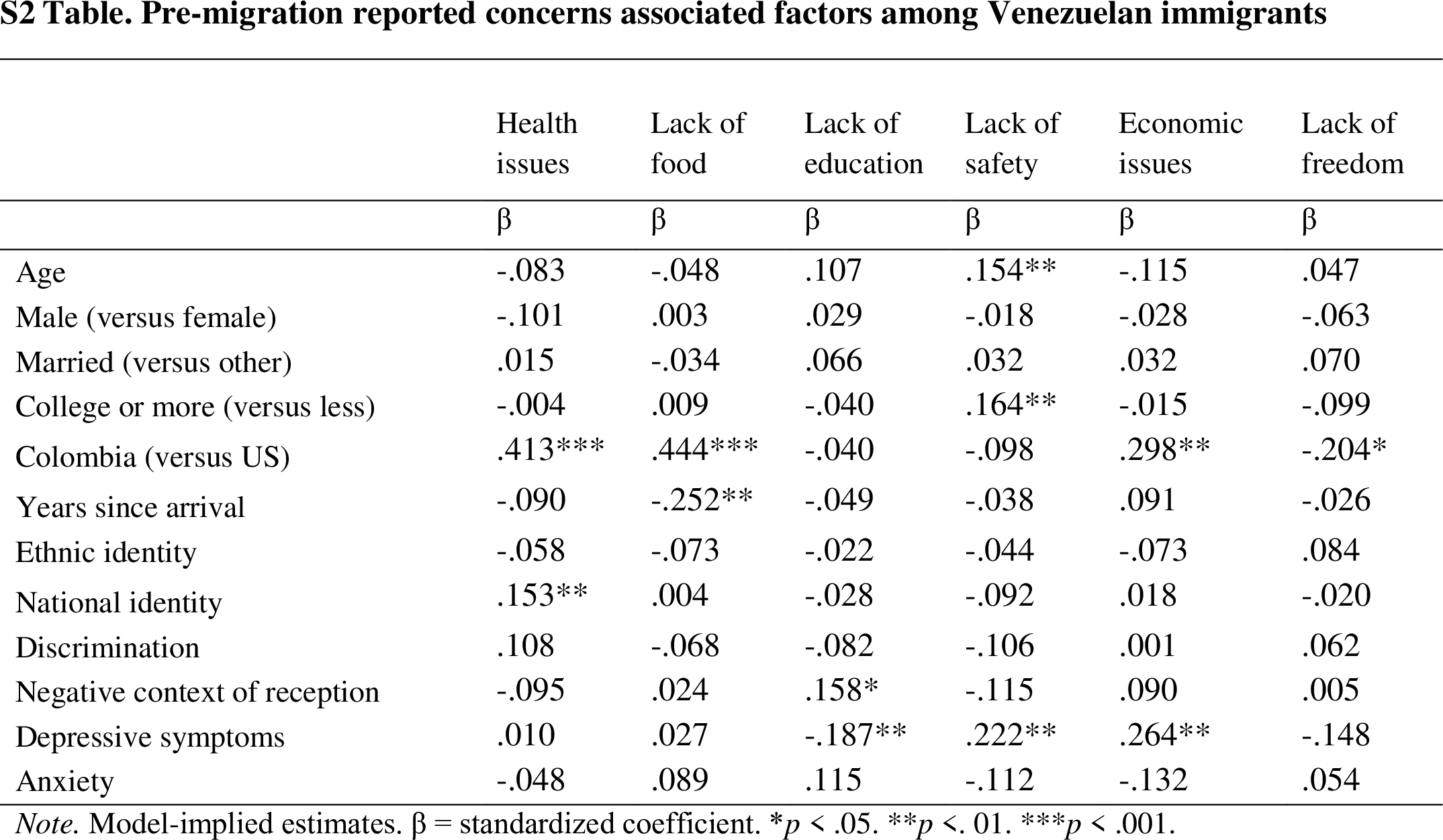

Supplement: S2 Table — (TIF) [file pone.0313215.s003.tif]

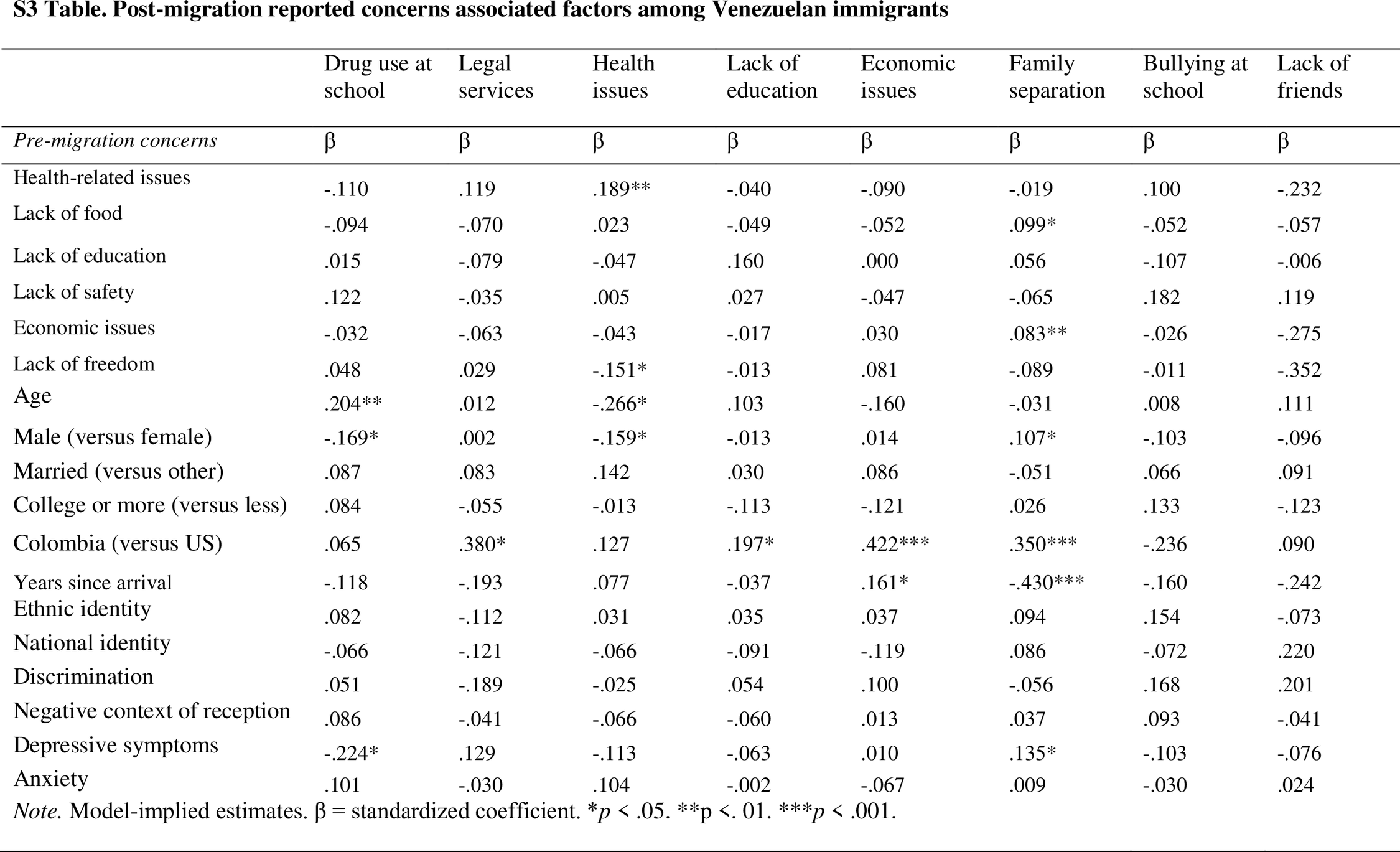

Supplement: S3 Table — (TIF) [file pone.0313215.s004.tif]

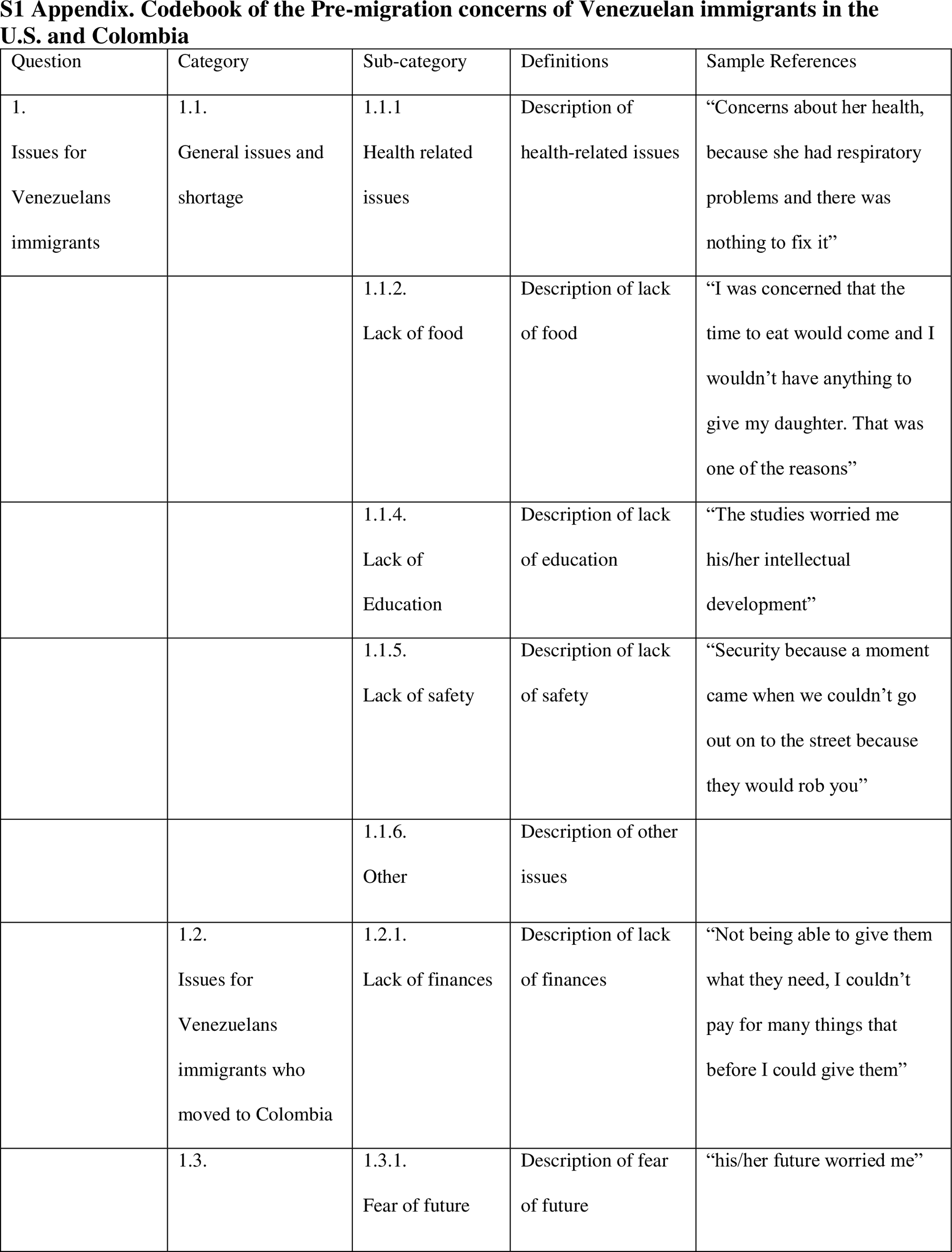

Supplement: S1 Appendix — (TIF) [file pone.0313215.s005.tif]

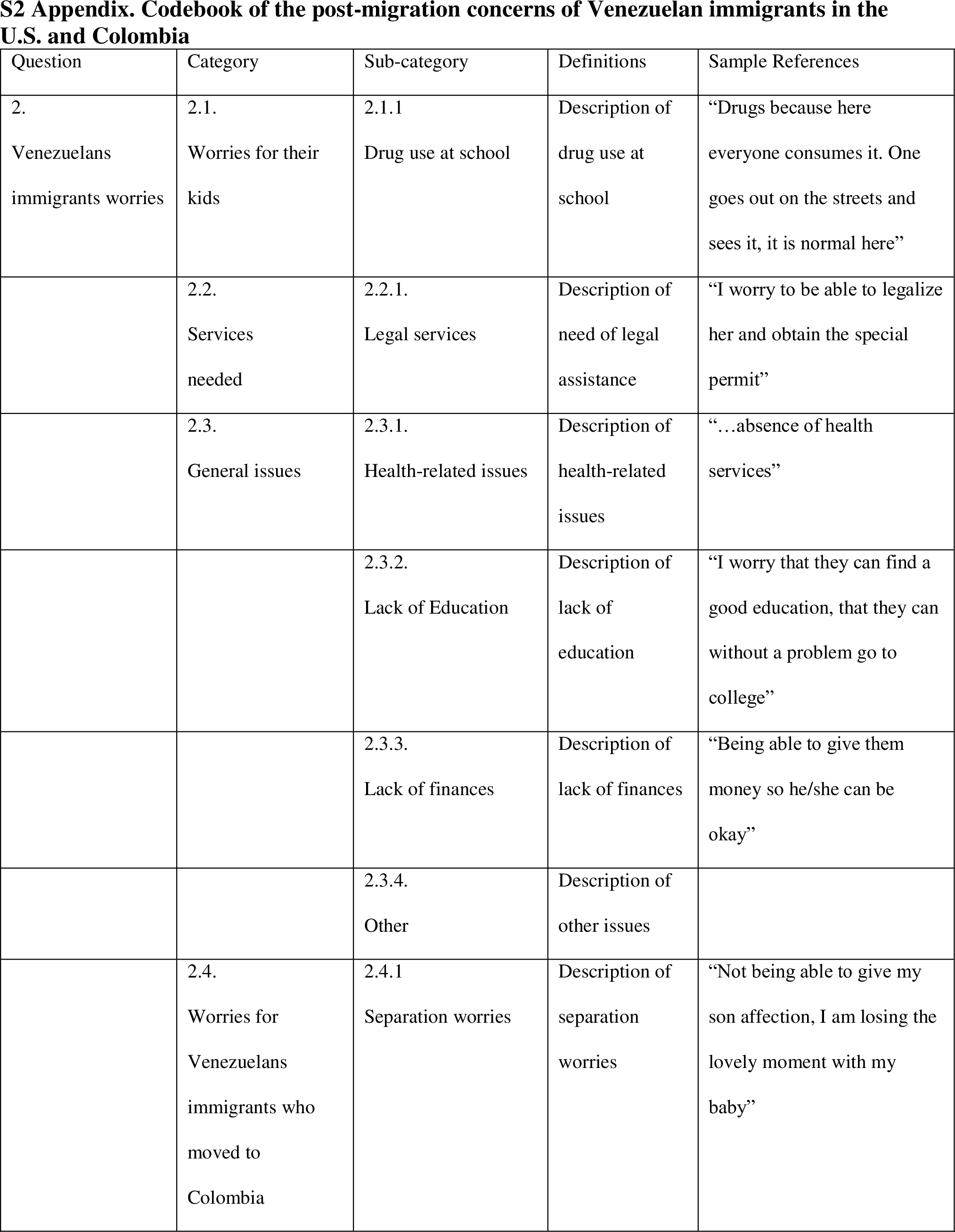

Supplement: S2 Appendix — (TIF) [file pone.0313215.s006.tif]
